# Exploring the immune-boosting and hepatoprotective potential of *Allium jesdianum* against cyclophosphamide-induced toxicity in mice: A promising approach for immunomodulation

**DOI:** 10.22038/ajp.2024.25258

**Published:** 2025

**Authors:** Alireza Rezaei, Bahareh Sadat Yousefsani, Ameneh Omidi, Kobra Shirani

**Affiliations:** 1 *Department of Toxicology, Faculty of Medicine Sciences, Tarbiat Modares University, Tehran, Iran*; 2 *Institute for Studies in Medical History, Persian and Complementary Medicine, Iran University of Medical Sciences, Tehran, Iran*; 3 *Department of Traditional Medicine, School of Persian Medicine, Iran University of Medical Sciences, Tehran, Iran*; 4 *Department of Anatomical Sciences, Faculty of Medical Sciences, Tarbiat Modares University, Tehran, Iran*

**Keywords:** Immunesuppression, Hepatoxicity, Allium jesdianum, Cellular immunity, Humoral immunity

## Abstract

**Objective:**

Cyclophosphamide (CTX) is a potent chemotherapy drug for treating cancer, but its use is limited due to its toxic effects on healthy human tissues. This study aimed to explore the* in vivo* immunomodulatory effects of *Allium jesdianum* on CTX-induced toxicity in Nordic Medical Research Institute (NMRI) mice.

**Materials and Methods:**

Hydroalcoholic extract of the whole plant of *A. jesdianum* (AJE) was obtained using the maceration technique, and its total phenolic and flavonoid contents were measured. Mice were orally administered with the extract at a dose of 200 mg/kg for 14 days, either as a standalone treatment or combined with an intraperitoneal injection of 20 mg CTX. The effects of the extract on body and relative organ weight, white blood cell (WBC) count**, **liver biochemical test, serum antibody titer hemagglutination (HA), delayed-type hypersensitivity reaction (DTHR), lymphocyte proliferation, cytokine production, and spleen and liver histopathological features were assessed.

**Results:**

AJE effectively restored various parameters in immunosuppressed mice, including body and organ weight, WBC counts, liver biochemical markers, HA, DTHR, lymphocyte proliferation ability, and cytokine production. Notably, AJE significantly stimulated lymphocyte proliferation, enhanced both cellular and humoral immunity, restored the levels of interferon (IFN)-γ and interleukin (IL)-4, and reversed the splenic white pulp atrophy in the immunosuppressed mice.

**Conclusion:**

Analyses have shown that AJE exerts protective effects on the immune system of CTX-treated animals by boosting both cellular and humoral immunity, with no observed hepatoxicity.

## Introduction

The immune system is a highly complex network of mechanisms that plays a vital role in protecting the body from disease-causing agents. It consists of innate and acquired immune responses which react differently to pathogens and invaders. Immunodeficiency disorders can arise from primary genetic defects that affect the function of the immune system, inhibiting immune cells or pathways. Secondary immunodeficiencies can result from environmental factors, infections, malnutrition, autoimmunity, or immunosuppressive drug treatments. In immunodeficiency, the ability of the immune system to fight infectious diseases is compromised or absent, leading to increased susceptibility to infection, morbidity, and even mortality (Chaplin, 2010; Nicholson, 2016). One category of immunosuppressive drugs consists of anticancer drugs. Many of these agents can inadvertently cause collateral damage to non-cancerous cells during therapy. This unintended damage may result in a range of adverse effects, including myelosuppression, hepatotoxicity, and gastrointestinal issues such as diarrhea (Sepahi et al., 2016). 

Cyclophosphamide (CTX) is a drug commonly used to treat cancer and autoimmune diseases, and as a preventive measure before transplantation. It belongs to the class of nitrogen mustard drugs and works by altering deoxyribonucleic acid (DNA) through alkylation. This disrupts protein synthesis by causing cross-linking of DNA and ribonucleic acid (RNA), ultimately leading to programmed cell death. However, one major limitation of CTX therapy is its toxic effects on healthy tissues in the body. This restriction on the dosage that can be administered impacts the treatment protocols, which in turn affects the overall quality of life for patients. Synthetic immunomodulatory agents, including CTX, can cause adverse reactions such as neutropenia, anorexia, and proteinuria. The side effects of CTX further complicate its use in medical treatments (Baumann and Preiss, 2001; Fraiser et al., 1991; Sistigu et al., 2011).

Medicinal plants have been used in healthcare for centuries and offer a potential alternative to conventional drugs due to their immunomodulatory properties and low toxicity (Shiehzadeh et al., 2022). *Allium* plants are part of the Amaryllidaceae family and have a wide range of medicinal properties, including antibacterial, antifungal, anti-diabetes, antihypertensive, blood cholesterol-lowering, and anti-atherosclerotic effects. The *Allium* genus, which includes garlic, onion, shallot, leek, chives, and scallions, has been found to have beneficial effects on the immune system and help alleviate chemotherapy symptoms (Abedi et al., 2024; Shiehzadeh et al., 2022)

*Allium jesdianum*, also known as “*Bowsor”*, “*Sarpa”*, or “*Bonsorkh”* in the local language, is an onion-like plant found in the western and southwestern highlands of Iran. This plant has been traditionally used in medicine and has been found to possess various medicinal properties. It has shown analgesic effects, can prevent platelet aggregation and kidney stone formation, and exhibits anticancer and neuroprotective activities. Despite the extensive use of *Allium* plants in traditional medicine and the known immunomodulatory properties of plants from the same genus, few studies study have been conducted to investigate the effects of this plant on the immune system (Ghoranipour et al., 2023; Kamranfar et al., 2023; Keshavarzipoor et al., 2024). 

The purpose of this study was to examine the hepatoprotective and immunomodulatory effects of *A. jesdianum* extract (AJE) in mice with CTX-induced toxicity. 

## Materials and Methods

### Preparation of plant sample

The *A. jesdianum* collected in spring 2021, from Bakhtiari Mountains was coded (A-0138) by the relevant expert at Jundishapur University, Ahvaz, Iran. One hundred grams of whole plant dried powder was extracted by 300 ml of 80:20 ethanol: water solvent using maceration method. The extract was concentrated by a rotary evaporator and freeze dryer. The extracts were dried in vacuum oven at 40°C for five days. Then, they were stored at 4°C until used (Shirani et al., 2023).

### Measurement of total phenolic compounds

A spectrophotometric analysis was performed to assess the total phenolic amount utilizing the Folin-Ciocalteu assay, with gallic acid employed as the standard described previously (Fattahi et al., 2014).

### Measurement of total flavonoid compounds

The total flavonoid amount was assessed utilizing a colorimetric assay with quercetin employed as the standard described previously (Chang et al., 2002).

### Animals

Nordic Medical Research Institute (NMRI) mice, aged 6 to 8 weeks (19 to 21 g), were obtained from the Faculty of Medical Sciences at Tarbiat Modares University, Tehran, Iran. Prior to starting any experiments, the mice were housed in polystyrene cages with five mice per cage for one week in a laboratory setting. The mice were kept under standard conditions (20-22°C, relative humidity of 35%, and a 12-hr light-dark cycle). Mice had unlimited access to food and water during the habituation period. All animal experiments conducted in this study received approval from the ethical committee of Tarbiat Modares University,Tehran, Iran. (approval number: IR.MODARES.AEC.1401.017).

### Doses and exposure schedules

Sixty male mice were divided into three groups for different experiments. In the first group, we conducted a histopathological evaluation of the spleen and liver, monitored changes in animal weight, assessed relative weights of different organs, and analyzed blood factors. A cell suspension was prepared from the spleen, and lymphocyte proliferation was measured using the MTT test. Additionally, levels of IFNγ and IL-4 were determined. In the second group, humoral immunity was assessed by measuring the antibody titer using the hemagglutination (HA) method.

In the third group, we investigated the function of the cellular immune system through the delayed-type hypersensitivity (DTH) response. 

Each group further divided into four subsets. The first subset received 200 mg/kg AJE orally for 14 days. The second subset received 200 mg/kg AJE orally for 14 days, combined with 20 mg/kg CTX (intraperitoneal (IP) injection) during the last five days. The third subset received 20 mg/kg CTX (IP) for five days, serving as the positive control group. The fourth subset received oral administration of normal saline for 14 days, representing the negative control group (Jalili et al., 2020; Keshavarzipoor et al 2023; Riahi-Zanjani  et al., 2015).

### Body and relative organ weight

To assess weight changes, the mice's weights were measured on the first day before receiving the initial dose. Following weight measurement on the 14^th^ day, two hours after administering the final dose, the animals were euthanized by severing the spinal cord. and the weight of the spleens and liver was recorded. The organs’ relative weight was calculated as organ weight/body weight.

### White blood cell count

Two hours after the final dose was given on the fourteenth day, a blood sample of 0.2 ml was collected from the retro-orbital plexus into dipotassium ethylene diamine tetraacetic acid (K_2_ EDTA) tubes. The total white blood cell (WBC) count and the ratio of different types of WBCs were determined.

### Liver biochemical tests

Blood samples from each mouse were individually collected in sterilized dry centrifuge tubes through cardiac puncture. The collected samples were then allowed to coagulate for 10 min at 37°C. Following coagulation, the clear serum was separated by centrifuging at 2500 rotations per minute (rpm) for 10 min and sent to a medical laboratory for biochemical estimations.

### Preparation of single-cell suspension

On the 14^th^ day, two hours after the last dose, the animals were killed by spinal cord transection. The spleen was then removed aseptically and suspended as previously described. The number of live cells was measured using trypan blue under a light microscope (Riahi-Zanjani  et al., 2015).

### Serum antibody titer hemagglutination (HA)

Serum antibody titer hemagglutination (HA) was done using Freund's complete adjuvant as described previously. Briefly, 1 mL of washed sheep red blood cells (SRBCs) was diluted to 5×10^8 using Freund's complete adjuvant, and on the 11th day via IP injection. After serum was collected, two-fold dilutions of the sera were prepared, and agglutination was assessed by mixing 25 µL of serum with 25 µL of SRBC suspension, incubating at 37°C for 1 hour to determine the antibody titer (Riahi-Zanjani  et al., 2015).

### Delayed-type hypersensitivity reaction (DTHR)

The DTHR test in this study followed a modified version of Fararjeh et al. assay described previously (Fararjeh et al., 2008). 

### Lymphocyte proliferation

A total of 100 𝜇l of spleen cell suspension was added to each well of a 96-well plate. Each well received either no mitogen, lipopolysaccharide (LPS), or phytohemagglutinin (PHA) at concentrations of 1 and 5 𝜇g/ml, respectively. The plate was then incubated at 37°C and 5% carbon dioxide (CO_2_) for 48 hr for cell attachment and stimulation. Following incubation, cell proliferation was assessed by the MTT (3-(4,5-dimethylthiazol-2-yl)-2,5-diphenyl-2H-tetrazolium bromide) assay. The proliferation index (PI) was calculated to evaluate the extent of cell proliferation:

PI = absorbance of stimulated spleen cells∕ absorbance of spleen unstimulated cells (Shirani et al., 2021).

### Production of cytokines

Cells were exposed to mitogens LPS or PHA and cultured for 48 hr at 37°C in a 5% CO_2_ environment. The cell-free supernatants were used to quantify the levels of interferon (IFN)-γ and interleukin (IL)-4, by available enzyme-linked immunosorbent assay kit (Karmania Pars Gene) according to the manufacturers' protocols (Shirani et al., 2021).

### Histopathological examinations

The spleen and liver were collected from each mouse and fixed in 10% formalin. Following mounting, 5-μm thick sections of these tissues were stained with hematoxylin and eosin (H and E) and analyzed via light microscopy (Shirani et al., 2018). 

### Statistical analysis

All data are presented as the mean ± SD and were analyzed using one-way ANOVA, followed by Tukey’s multiple comparison test using PRISM, version 6.00 (GraphPad Software Inc., San Diego, CA, USA). A p<0.05 was considered statistically significant. 

## Results

**Amount of total phenolic compounds in the extract**

The total phenolic content of the extract was 157.6 mg/1 gram of dry extract.

**Amount of total flavonoid compounds in the extract**

The total flavonoid compounds amount of the extract was 114.7 mg/1 gram of dry extract.

**Effect on mouse body and relative organ weight**

As shown in Table 1, the group treated with CTX showed a considerable decrease in body weight compared to the control (p<0.05). The relative spleen weight of the group treated with AJE alone was notably higher than the control (p<0.01). However, the group treated with CTX displayed a significant decrease in spleen weight compared to the control group (p<0.05). Additionally, the AJE 200 mg/kg significantly increased spleen weight and relative spleen weight compared to the CTX group (p<0.01). Furthermore, the group treated with CTX showed a significant decrease in liver weight compared to the control group (p<0.05). Moreover, the co-administration of AJE 200 mg/kg resulted in a significant increase in liver weight and relative liver weight compared to the CTX group (p<0.01).

**Effect on liver biochemical factors**

Mice treated with CTX alone developed significant hepatocellular damage, as evidenced by an increase in serum biomarkers ALP (alkaline phosphatase), ALT (alanine transaminase), AST (aspartate aminotransferase), and total bilirubin when compared to the control group. Co-administration of CTX with AJE 200 mg/kg resulted in a substantial decrease in the levels of AST, ALT, ALP, and total bilirubin that was nearly equivalent to the control group (Table 2).

**WBC counts**

Compared to the control group, AJE 200 mg/kg demonstrated the ability to alleviate the reduction of WBC, lymphocytes, and monocytes induced by CTX (20 mg/kg) (Table 3).

**Table 1:** The protective effect of AJE on body weight and relative organ weight in mice.

**CTX**	**AJE** **+ CTX**	**AJE**	**NS**	
28.1±2.8	28.1±2	27.5±2.5	28±2	Body weight (g) Before
29±3.2^*^	33±3	34±1.5	34.5±3.5	Body weight (g) After
1.05 ±0.09^**^	1.5 ±0.19^##^	1.75 ±0.17	1.65 ±0.09	Liver
3.6^**^	4.5^##^	5.4^*^	4.7	Relative weight %BW
0.26 ±0.06^*^	0.47 ±0.04^##^	0.54 ±0.06^**^	0.36 ±0.01	Spleen
0.89^*^	1.4^##^	1.5^**^	1.04	Relative weight %BW

Data are the mean±SD. *p<0.05 and **p<0.01: significant changes compared to the control group (NS). +p<0.05 and ++p<0.01 compared to the CTX group. AJE (*A. jesdianum* extract), CTX (Cyclophosphamide), NS (Normal Saline).

**Table 2:** Effects of subacute oral exposure of mice to AJE for 14 days on white blood cell counts in cyclophosphamide-exposed mice.

**CTX**	**AJE** **+CTX**	**AJE**	**NS**	
1.65±0.72^**^	3.05±0.12^+++^	5.82±0.20^***^	3.25±0.43	WBC (10^4^/µl)
1.07±0.17^**^	2.10±0.11^++^	4.07±0.1^***^	2.25±0.42	LYM (10^4^/µl)
0.33±0.13^*^	0.78±0.21^+^	1.41±0.15^*^	0.75±0.15	MONO (10^4^/µl)
0.17±0.06	0.29±0.21^+^	0.32±0.17^*^	0.24±0.01	NEU (10^4^/µl)

Data are the mean±SD. *p<0.05, **p<0.01 and ^***^p<0.001: significant changes compared to the control group (NS). +p<0.05, ++p<0.01, and +++p<0.001 compared to the CTX group. AJE (*A. jesdianum* extract), CTX (Cyclophosphamide), NS (Normal Saline), WBC (White Blood Cells), LYM (Lymphocytes ), MONO (Monocracy), NEU (Neutrophil)

**Table 3:** Effects of subacute oral exposure of mice to AJE for 14 days on biochemical parameters in cyclophosphamide exposed mice.

**CTX**	**AJE** **+CTX**	**AJE**	**NS**	
94.65±22.72^***^	73.05±10.12^+++^	65.02±12.20	60.42± 15.39	ALT (U/L)
107±31.17^***^	72.12±21.11^++^	74.27±18.1	70.85± 20.87	AST (U/L)
100.33±23.13^***^	78±12.11^+^	73.41±13.1	75±28.15	ALP (U/L)
0.67±0.02^*^	0.39±0.21^+^	0.33±0.07	0.30 ±15.01	Total Bilirubin (mg/dl)

Data are the mean±SD. *p<0.05 and ^***^p<0.001: significant changes compared to the control group (NS). +p<0.05, ++p<0.01, and +++p<0.001 compared to the CTX group.

AJE (*A. jesdianum* extract), ALP (Alkaline Phosphatase), ALT (Alanine Transaminase), AST (Aspartate Aminotransferase), CTX (Cyclophosphamide)

**Spleen cell number**

**Figure 1:**
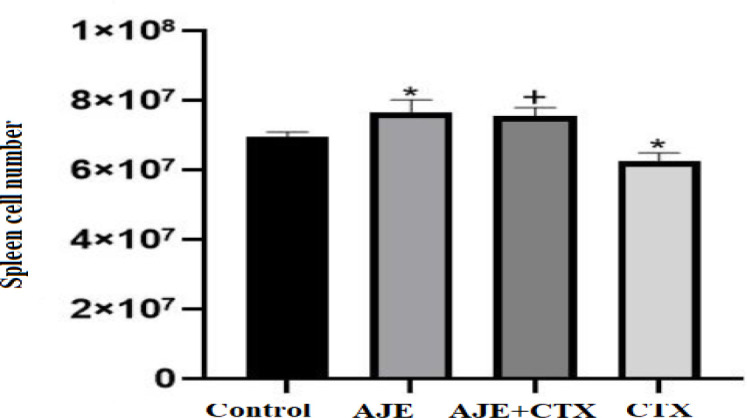
The protective effect of AJE exposure on spleen cell number in CTX exposed mice. Data are presented as the mean±SD (n=5). *p<0.05 compared to the control group (NS), ++p<0.01 compared to the CTX group. Data were analyzed through one-way ANOVA coupled with Tukey-Kramer multiple comparisons test. AJE (A. jesdianum extract), CTX (Cyclophosphamide), NS (Normal Saline).

Figure 1 demonstrates a striking growth in splenocyte number in mice treated with AJE 200 mg/kg compared to the control (p<0.05). Conversely, administration of CTX led to a marked decrease in splenocyte numbers, which was reversed by AJE compared to the CTX-only group (p<0.05).

**HA titer**

**Figure 2:**
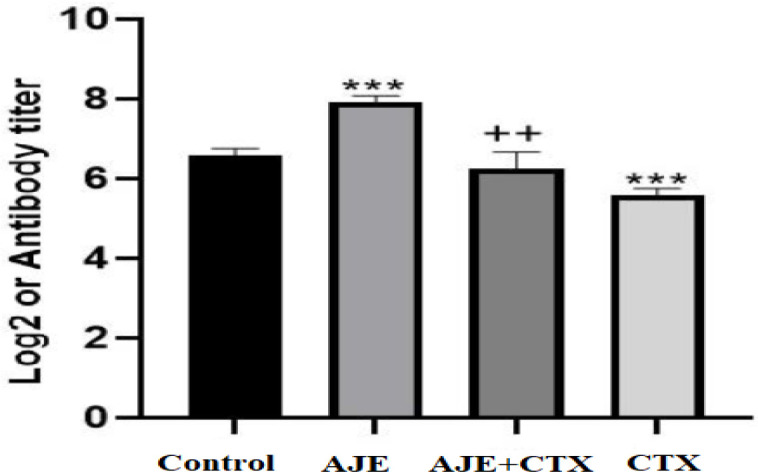
The protective effect of AJE on hemagglutination (HA) titer in CTX exposed mice. Data are presented as the mean±SD (n= 5 animals). **p<0.01 and ***p<0.001 compared to the control group (NS), ++p<0.01 compared to the CTX group. Data were analyzed through one-way ANOVA coupled with Tukey-Kramer multiple comparisons test. AJE (A. jesdianum extract), CTX (Cyclophosphamide), NS (Normal Saline).

Analysis of serum antibody titer revealed a significant decrease in antibody production in CTX-treated mice compared to the control group (p<0.001). Conversely, the group treated with AJE exhibited a significant increase in antibody production compared to the control (p<0.001). Furthermore, the mice receiving the combination of AJE and CTX demonstrated significantly higher antibody production compared to the CTX-only group (p<0.01) (Figure 2).

**DTHR**

**Figure 3:**
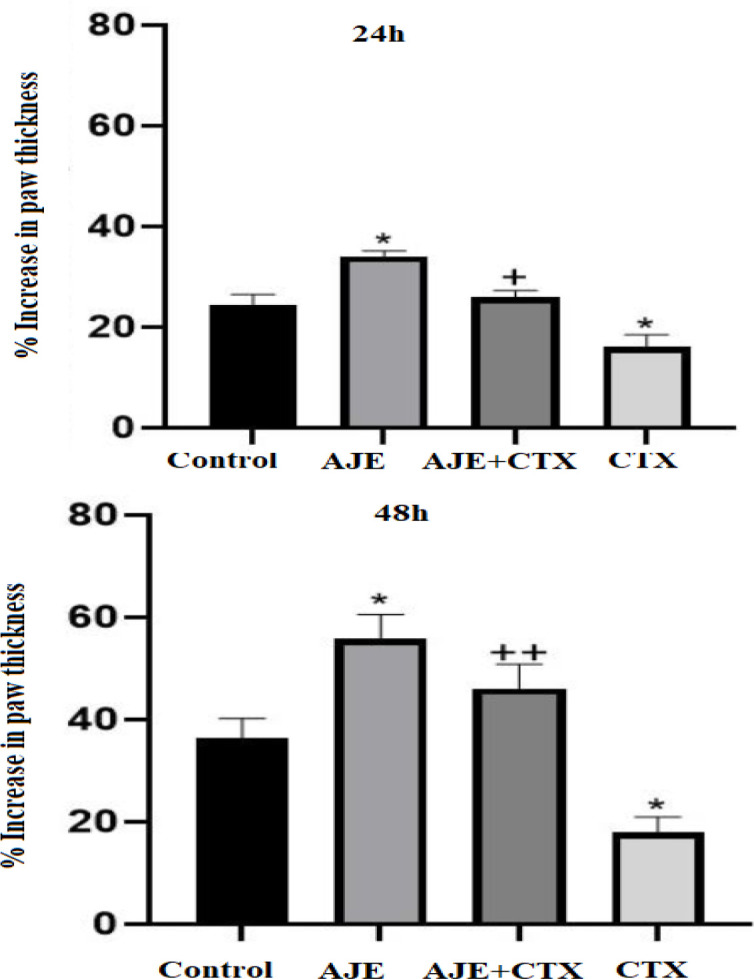
The protective effect of AJE on delayed-type hypersensitivity (DTHR) response after 24 hr (A) and 48 hr (B) in CTX exposed mice. Data are presented as the mean±SD (n= 5). *p<0.05 compared to control group (NS), + p<0.05 and ++ p<0.01 compared to CTX group. Data were analyzed through one-way ANOVA coupled with Tukey-Kramer multiple comparisons test. AJE (A. jesdianum extract), CTX (Cyclophosphamide), NS (Normal Saline).

Figure 3A illustrates a statistically significant decrease and increase in the CTX and AJE group compared to the control after 24 hr, respectively (p<0.05). Additionally, the AJE+ CTX group exhibited a significant increase in the DTHR compared to the CTX group. After 48 hr (Figure 3B), there was a significant elevation in the DTHR in the groups that received a combination of AJE and CTX compared to the control (p<0.01). 

**Lymphocyte proliferation**

**Figure 4:**
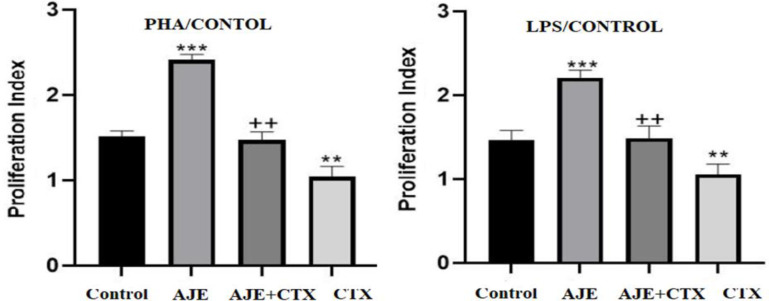
The protective effect of AJE on the lymphocyte proliferation response to LPS (A) and PHA (B) in mice exposed to CTX. Data are presented as the mean±SD (n=5). **p<0.01 and ***p<0.001 compared to the control group (NS), ++p<0.01 compared to the CTX group. Data were analyzed through one-way ANOVA coupled with Tukey-Kramer multiple comparisons test. AJE (A. jesdianum extract), CTX (Cyclophosphamide), LPS (Lipopolysaccharide), NS (Normal Saline), PHA (Phytohemagglutinin).

In Figure 4, it is evident that AJE significantly enhanced cell proliferation in response to PHA and LPS when compared to the control (p<0.001). Conversely, the CTX group exhibited a significant decrease in cell proliferation compared to the control (p<0.01). Furthermore, administration of AJE effectively mitigated the CTX-induced suppression of cell proliferation observed in the CTX group (p<0.01).

**Effect on the secretion IFN-γ and IL-4**

Mice exposed to CTX exhibited significantly reduced IFN-𝛾 and IL-4 concentrations. However, according to Table 4, the administration of AJE restored the IFN-𝛾 and IL-4 levels in the immunosuppressed mice. 

**Histopathology**

**Figure 5:**
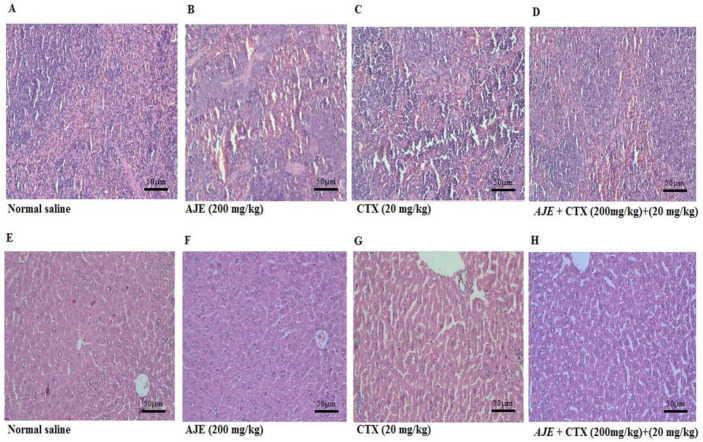
The protective effect of AJE exposure on CTX -induced histological damages in the spleen and liver of mice; spleen and liver sections were stained using the hematoxylin–eosin method (200X). AJE (A. jesdianum extract), CTX (Cyclophosphamide), NS (Normal Saline).

The spleen was evaluated for white pulp atrophy (or hyperplasia), red pulp: white pulp (Table 5). As observed in Figure 5A, there are no changes in the normal saline group. Analyses revealed that CTX induced splenic white pulp atrophy and an increase in the red: white pulp ratio, which was reversed by AJE (Figure 5C and 5D).

Histological analysis of the liver confirmed the outcomes of blood enzyme 

testing and demonstrated the hepatoprotective properties of the AJE. Mice given CTX alone exhibited Neutrophil (PMN) infiltration in the hepatic tissue, which is indicative of inflammation, as well as disorganized hepatic plates. However, AJE was able to restore the hepatic tissue, as shown in Figure *(*Figure 5G and 5H).

**Table 4:** Effects of subacute oral exposure of mice to AJE for 14 days on cytokines concentrations in cyclophosphamide exposed mice.

**CTX**	**AJE** **+ CTX**	**AJE**	**NS**	
195 ± 19^***^	239±21^+++^	249±15	269±13	IFN-𝛾 (pg/ml)
79±19^***^	121± 13^++^	157 ±12	159± 21	IL-4 (pg/ml)

Data are the mean±SD. ^***^p<0.001: significant changes compared to the control group (NS). ++p<0.01 and +++ p<0.001 compared to the CTX group. AJE (A. jesdianum extract), CTX (Cyclophosphamide), NS (Normal Saline), IFN-𝛾 (Interferon gamma), IL-4 (Interleukin 4 ).

**Table 5:** The protective effect of AJE exposure on CTX -induced histological damages in the spleen and liver of mice.

**Organ**	**Histological damages**	**NS**	**AJE**	**AJE** **+CTX**	**CTX**
**L**	Inflammatory reaction including Neutrophil (PMN) infiltration	-	-	+	+++
Liver	Disorganized hepatic plates	-	-	+	++
	Cell death (necrosis, apoptosis)	-	-	+	++
	White pulp atrophy	-	-	+	++
Spleen	Capsular-trabecular change	-	-	+	+
	Red pulp: white pulp ratio	Normal	Normal	Increase	Increase

−: No changes observed, +: minimal observable changes, ++: moderate detectable changes, and +++ severe detectable changes. AJE (*A. jesdianum* extract), CTX (Cyclophosphamide), NS (Normal Saline).

## Discussion

The *Allium* plants which are widely distributed worldwide, can increase host immune responses to various diseases by regulating immunity (Arreola et al., 2015). This finding has drawn considerable attention to investigating whether AJE could be a promising natural immunomodulator. 

In this study, a murine model of CTX-induced immunosuppression was employed 

intraperitoneally administered with CTX (20 mg/kg) from the beginning of the experiment for five days. The results obtained from this study demonstrated that the mice treated with CTX showed significantly reduced body weight and immune organ indices, WBC count, spleen cell number, lymphocyte proliferation ability, down-regulated cytokines, and suppression of DTHR and HAR, which reversed by AJE exerting its protective effect against CTX- immunosuppression. The pretreatment refers to the administration of AJE to mice before exposure to CTX. This approach aims to enhance immune function and protect the liver from toxicity induced by CTX.

Medicinal plants are gaining attention for their bioactive compounds with positive health effects. However, safety concerns and toxicity limitations restrict their usage. Liver injury is a common sign of toxicity in medicinal herbs. Blood biomarkers like ALT, AST, ALP, and bilirubin are used to assess liver function, with elevated levels indicating hepatic damage. In this study, AJE demonstrated significant hepatoprotective effects against CTX-induced liver injury. In normal mice, AJE pretreatment did not show any harmful effects, while CTX administration increased liver function markers such as ALT, AST, ALP, and bilirubin levels. However, AJE pretreatment successfully reduced the elevated levels of these markers, indicating its hepatoprotective ability. Histopathological examination also confirmed the hepatoprotective effects of the treatment, showing improved liver architecture and reduced inflammation. These findings align with previous studies highlighting the hepatoprotective potential and phytochemical composition of the tested extract (Sohrabinezhad et al., 2019; Kalantaria et al., 2019). The hepatoprotective activity of AJE may be attributed to its antioxidant properties, which are maybe linked to sulfur-containing constituents found in *Allium* species such as diallyl sulfide, S-methyl cysteine 2,5-dimethylthiophene, and 1-propylmercaptan. These sulfur-containing constituents have been shown to increase total thiol (TTG) and glutathione (GSH) levels, as well as exhibit antioxidative and antimutagenic properties (Sohrabinezhad et al., 2019; Kalantaria et al., 2019).

As shown in Figure 1, exposure to CTX resulted in a reduction in spleen mass, potentially due to the direct cytolytic effect of CTX on splenocytes. Conversely, treatment with AJE led to an increase in spleen weight, possibly due to an augmentation in the population of various lymphocytes within these organs (Figure 2, 3). Our findings are consistent with the another study which showed that *Allium* species could boost immune system cells (Mirabeau and Samson, 2012).

Lymphocyte proliferation is the initial stage of an effective immune response, leading to the production of effector lymphocytes (Desforges et al., 2018). These effector lymphocytes play an important role in eliminating the present antigen, while memory lymphocytes are generated simultaneously. Memory lymphocytes are essential for combating the same antigen if the host reencounters it in the future. This memory function ensures that subsequent responses to the antigen are swift and efficient (Ratajczak et al., 2018). In this study, it was found that AJE has a significant impact on enhancing the proliferation of T lymphocytes stimulated by PHA and B-lymphocytes stimulated by LPS in mice treated with CTX, which causes immunosuppression. These finding suggests that AJE exerts protective effects on CTX-induced immunosuppressed mice by boosting both cellular and humoral immunity.

T lymphocytes play a crucial role in defending the host against microbial pathogens by producing various cytokines. These immune responses can be categorized into type 1 T helper (Th1) and type 2 T helper (Th2) cell responses, with distinct functions in the immune system. Th1 lymphocytes primarily contribute to cellular immune responses by producing cytokines such as IFN-γ. On the other hand, Th2 lymphocytes stimulate the humoral response, support B cell proliferation, and trigger antibody production by secreting cytokines like IL-4 (Eagar and Miller, 2019; Moore et al., 2001). As shown in Table 3, the increase in both IFN-γ and IL-4 levels in the AJE + CTX groups indicates that the plant exhibits protective effects through both Th1 and Th2 pathways. IFN-γ, a key cytokine produced by Th1 cells, is primarily involved in promoting cell-mediated immune responses, enhancing the activity of macrophages, and supporting the differentiation of CD8+ T cells. On the other hand, IL-4 is crucial for the differentiation of naive T cells into Th2 cells, which enhance humoral immunity by promoting B cell activation and antibody production. This dual activation suggests that the extract from the plant not only strengthens the cell-mediated immune response but also promotes antibody production and adaptive immunity, reflecting a balanced immune response (Shirani et al., 2021). Such a phenomenon may provide a more comprehensive protective mechanism against various pathogens and diseases, making AJE a promising candidate for immunomodulatory therapies.

Serum antibody titer hemagglutination (HA) is used to detect and titrate antibodies developed against an antigen. Antibodies are essential components of humoral immunity playing crucial role in activating the complement system, specifically binding to antigens, and performing vital functions such as neutralizing infectious agents and preventing bacterial attachment and/or entry into host cells (Czakó et al., 2012, Kaufmann et al., 2017). According to the findings of lymphocyte proliferation assay, the current study observed a notable decline in antibody production against SRBC in the CTX group which was reversed by AJE indicating its protective effects on humoral immunity.

DTHR is a significant expression of cell-mediated immunity in living organisms. DTHR is triggered by immunization or infection with intracellular parasites, and it relies on antigen-specific T cells. Immune response involves the production of activating and chemotactic cytokines, an increase in vascular permeability, and the recruitment of non-specific effector cells to the reaction site (Kalish and Askenase, 1999). The results of the DTHR showed a robust immune response in mice receiving AJE. Due to the fact that by measuring the DTHR, it is feasible to indirectly understand the stimulation of Th2, Th1, and type 17 T helper (Th17) cells, it seems that AJE*,* like the other *Allium* species, may affect the balance of Th1/Th2 and even affect the activity of Th17 cells. Different studies have shown that flavonoids enhanced the DTHR and stimulated the secretion of IFNγ from Th1 cells (Ioannone et al., 2013; Nair et al., 2002). As the amount of flavonoids in the AJE was 114.7 mg/g of dry extract based on quercetin, the observed strengthening effect can be attributed to these compounds. Moreover, sulfur compounds are the most common constituents of AJE. These compounds, including S-allyl mercapto cysteine, dipropyl trisulfide, and allicin have demonstrated potential immunomudulatory effects by changing the balance of Th17/ Treg cells in favor of Treg (regulatory T) cells (Amiri, 2007; Asemani et al., 2019)

According to the findings of this study, the AJE has the potential to recover the immune system from CTX-induced immunosuppression. This effect is achieved by regulating cytokine secretion levels and strengthening the immune system. The protective impact of AJE on the immune system may be attributed to its polyphenol, flavonoid, and sulfur compounds content. To gain a deeper understanding of how the AJE affects various immune system mechanisms, further extensive research is required.
